# A bZIP transcription factor, PqbZIP1, is involved in the plant defense response of American ginseng

**DOI:** 10.7717/peerj.12939

**Published:** 2022-03-08

**Authors:** Shanshan Yang, Xiaoxiao Zhang, Ximei Zhang, Yanmeng Bi, Weiwei Gao

**Affiliations:** 1 Institute of Medicinal Plant Development, Chinese Academy of Medical Sciences and Peking Union Medical College, Beijing, China; 2 College of Agriculture, Guangxi University, Nanning, China; 3 Institute of Food Science and Technology, Chinese Academy of Agricultural Sciences, Beijing, China; 4 School of Environmental and Municipal Engineering, Tianjin Chengjian University, Tianjin, China

**Keywords:** American ginseng, *Panax quinquefolius* L., PqbZIP1, Root rot disease, *Fusarium solani*, Immune response, Disease resistance genes

## Abstract

American ginseng (*Panax quinquefolius* L.) is a perennial medicinal plant that has a long usage history in China. However, root rot, which is mainly caused by *Fusarium solani* can severely reduce the yield and quality of American ginseng, but no disease-resistant variety of American ginseng exists, and the resistance against this disease is not yet well understood. Thus, it is very urgent to analyze the interaction mechanism regulating the interactions between American ginseng and *F. solani* to mine disease resistance genes. Using transcriptome data and quantitative polymerase chain reaction (qPCR), we screened the transcription factor PqbZIP1 in response to induction by chitin. Yeast self-activation and subcellular localization experiments proved that PqbZIP1 showed transcriptional activity and was localized in the plant nucleus. In addition, qPCR showed that the highest relative expression level was in the roots, wherein chitin and *F. solani* inhibited and activated the expression of *PqbZIP1*, respectively, in American ginseng. Additionally, PqbZIP1 significantly inhibited the growth of the *Pseudomonas syringae* pv. *tomato* D36E strain in *Nicotiana benthamiana*, where expressing *PqbZIP1* in *N. benthamiana* increased the jasmonic acid, salicylic acid, and abscisic acid content. Furthermore, *PqbZIP1* expression was continually increased upon inoculation with *F. solani*. Hence, this study revealed that the PqbZIP1 transcription factor might mediate multiple hormonal signaling pathway to modulate root rot disease resistance in American ginseng, and provided important information to breed disease-resistant American ginseng.

## Introduction

American ginseng (*Panax quinquefolius* L.) is a perennial medicinal plant. Its root has been used in China as an herb for hundreds of years and has been used in a wide range of treatments for diabetes, tumors, cardiovascular enhancement, the central nervous system, and immunomodulation (Yuan et al., 2010; Jiao et al., 2019; Qi, Wang & Yuan, 2010). American ginseng plants require a shady environment and more than 4 years of growth before harvesting. Because of this, American ginseng is susceptible to soil-borne diseases such as root rot disease that is mainly caused by *Fusarium solani* (Jiang et al., 2019; Walsh et al., 2019). Root rot disease causes a decline in yield and limits the sustainable development of American ginseng in the areas of its production.

Plants are attacked by various pathogens in nature. As part of their innate immune system, plants recognize these pathogens by virtue of pathogen-associated molecular patterns (PAMPs) (Gong, Wang & Li, 2020; Nurnberger et al., 2004; Boller & He, 2009; Couto & Zipfel, 2016; Pusztahelyi, 2018). PAMPs include molecules such as flagellin (Gomezgomez, 2004) and chitin (Gong, Wang & Li, 2020). The recognition of pathogens by PAMPs results in the induction of various pathogen defense pathways (such as plant hormones) and pathogenesis-related proteins (such as chitinase). Purified chitin oligomers are a conserved component of fungal cell walls whose application in plants can elicit various defense-related reactions (Libault et al., 2007). Ramonell et al. (2005), Ramonell et al. (2002) reported that many *Arabidopsis thaliana* genes were induced by chitin, and mutations in some genes identified responsive to chitin resulted in increased susceptibility to the powdery mildew fungal pathogen *Erysiphe cichoracearum*. Yamaguchi et al. (2017) reported that *A. thaliana* could recognize fungal chitin through CERK1 kinase, triggering the phosphorylation of CERK1, and thereby activating defense signal transduction. Moreover, the *Verticillium nonalfalfae* ChtBP effector protein could inhibit the reactive oxygen species (ROS) burst that was caused by chitin in hop plants, which interfered with the immune response (Volk et al., 2019). A comparison of the response of wild-type *Arabidopsis* with various mutants with blocked ET, SA, and JA signaling pathways showed that the elicitation of the initial response to chitin was independent of these pathways (Zhang et al., 2002). In addition, chitin pretreatment also reduces the susceptibility of the plants to subsequent fungal pathogen challenge (Tanabe et al., 2006). Thus, chitin elicitation plays an important role in plant defense to fungi, and using chitin instead of fungi to induce an immune response is an effective method for screening immune-related genes.

Chitin can affect resistance gene expression *via* transcription factors (Libault et al., 2007), which have been shown to play a key role in a plant’s resistance to pathogens (Singh, Foley & Oñate-Sánchez, 2002). The discovered transcription factors of disease resistance genes mainly include: the TGA type in the bZIP family, the ERF-type transcription factor, and the WRKY family in the zinc finger protein. Whereas Xu et al. (2019) found that bZIP60 could increase the resistance of *Nicotiana attenuata* to *Alternaria alternate*. In addition, overexpressing *MebZIP3* and *MebZIP5* in cassava improved disease resistance against the cassava bacterial blight, and silencing the two genes resulted in a disease-sensitive phenotype (Li et al., 2017). Moreover, pepper CAbZIP1 functioned as a possible regulator to enhance disease resistance, where *A. thaliana* overexpressing *CAbZIP1* enhanced its resistance to *Pseudomonas syringae* pv. *tomato* DC3000 (Lee et al., 2006). Additionally, in *Nicotiana benthamiana*, the mitogen-activated protein kinase could phosphorylate WRKY8, which increased its DNA binding activity to the cognate W-box sequence, and then activated the expression of the downstream genes, thereby enhancing the ability to resist *Phytophthora infestans* and *Colletotrichum orbiculare* (Ishihama et al., 2011). However, the transcriptional regulatory mechanism underlying disease resistance genes in American ginseng is still unclear.

Furthermore, the plant resistance response induced by pathogenic fungi is often accompanied by ROS, callose deposition, and hormone levels regulated by transcription factors. For example, an infection by *Alternaria tenuissima* led to an increase in the endogenous jasmonic acid content of *Paeonia lactiflora*, and when the *PlWRKY65* gene was silenced, the jasmonic acid content decreased and the resistance of *P. lactiflora* to *A. tenuissima* was significantly weakened (Wang et al., 2020). In addition, the R2R3-MYB transcription factor MdMYB73 confered increased resistance to the fungal pathogen *Botryosphaeria dothidea* in apples *via* the salicylic acid pathway (Gu et al., 2021). Further, the negative regulation of ABA signaling by WRKY33 was critical for *Arabidopsis* immunity against *Botrytis cinerea*. Thus, identifying genes that are associated with the hormone signaling pathway is one method of mining resistance genes. Although several genes associated with the hormone signaling pathway have been shown to enhance plant immune response, few studies have been performed on American ginseng.

In this study, chitin was used to stimulate the immune response of American ginseng, which was combined with transcriptome sequencing technology to mine genes that were closely related to the immune response. From this, we successfully identified the transcription factor PqbZIP1, which responded to the chitin treatment. If *PqbZIP1* could be stimulated by *F. solani*, was detected by quantitative polymerase chain reaction (qPCR). Furthermore, we proved that PqbZIP1 could activate plant defense and prevent pathogen parasitism by modulating multiple hormone signaling pathways in plants. This study provides a simple interpretation of how the PqbZIP1 transcription factor can affect plant immunity. Moreover, this work can broaden our knowledge of the resistance mechanisms of American ginseng.

## Materials and Methods

### Fungal strain, plant materials and RNA extraction

Fungal strain 4171 was stored in our lab after being isolated from American ginseng roots with root rot symptoms. Two-year-old healthy and fresh American ginseng roots were obtained from a commercial farm in Weihai, China, in March 2020. Five similarly sized and healthy roots were first washed and soaked in chitin (100 µg/ml) at 25 °C for 1 h, quickly frozen by liquid nitrogen, and finally stored at −80 °C until further use. At the same time, water-treated roots were used as a negative control, where each treatment was performed in triplicates, with one treatment containing five roots. Total RNA was extracted using the TRIzol RNA extraction reagent (Invitrogen, Waltham, MA, USA) and the residual DNA was removed by DNase I (Takara, Japan). The RNA was first assessed using 2% agarose gels and then the total RNA was quantified with a NanoDrop 2000 (Themo Fisher Scientific, Waltham, MA, USA) for the construction of the RNA sequencing libraries. RNA integrity was assessed using the Agilent Technologies 2100 Bioanalyzer system (Santa Clara, CA, USA) with an RNA integrity number greater than 7 being used as the cutoff.

### Identification of strain 4171

We identified strain 4171 according to its colony appearance, Koch’s rules, and the maximum likelihood tree of *Fusarium* spp. based on a combination of *ITS* and *tef-1α* genes. We used the CTAB plant tissue DNA extraction kit for fungal genomic DNA extraction and amplification of the *ITS* sequence (White et al., 1990) and *tef-1a* sequence (O’Donnell et al., 1998).

### PacBio Iso-Seq library preparation and sequencing

American ginseng does not yet have a complete genome sequence to reference, so full-length transcriptome sequencing was adopted in the present study as a reference. The six individual samples were mixed together as one sample, which was pooled for the PacBio Iso-Seq library construction (Pacific Biosciences of California, Inc, Menlo Park, CA, USA). The full-length cDNAs were synthesized using the SMRTer PCR cDNA Synthesis Kit (Biomarker, Beijing, China) and the library was constructed using the SMRTbell Template Prep Kit (Pacific Biosciences of California, Inc, Menlo Park, CA, USA). Finally, the SMRT cells (1–6 kb) were sequenced on the PacBio Sequel II platform (Pacific Biosciences of California, Inc, Menlo Park, CA, USA).

### Illumina cDNA library construction and sequencing

Oligo (dT) magnetic beads were used to enrich the poly (A) mRNA from the six samples where the mRNA was fragmented into small pieces. Using these short fragments as templates, the first-strand cDNA was synthesized and used to synthesize the second-strand cDNA (Jin et al., 2017). Next, the AMPure XP beads (Beckman Coulter, USA) were used to purify these short double cDNA fragments. These fragments were ligated using Illumina paired-end adaptors and purified with AMPure XP beads after the end reparation and A-tailing. Then, the DNA fragments with the adapter molecules on both ends were enriched and the final cDNA library was created (He et al., 2019). Finally, the libraries were sequenced using the Illumina NovaSeq 6000 sequencing platform (Biomarker Technologies Corporation, Beijing, China).

### PacBio data analysis

First, the raw reads were processed into error-corrected reads of insert using the Iso-Seq pipeline. Next, full-length, non-chimeric transcripts were determined by searching for the polyA tail signal and the 5′ and 3′ cDNA primer in error-corrected reads of insert. Moreover, ICE (Iterative Clustering for Error Correction) was used to obtain the consensus isoforms and the full-length consensus sequences from ICE were polished using Quiver (Gordon et al., 2015). High quality full-length transcripts were classified by the criteria of post-correction accuracy above 99%. Then, all isoforms were corrected (Hackl et al., 2014). Finally, the longest isoform from each cluster was regarded as the transcript, and then corrected by the CD-HIT software (v4.6).

### Structure analysis

Variable splicing candidate events are predicted based on the transcripts of three generations of non-parametric transcriptomes after redundancy removal. In the absence of a reference genome, all transcripts were pairwise compared to determine that the gap satisfying the conditions was the result of variable splicing. We directly used the Iso-SeqTM data to run the all-vs-all basic local alignment search tool (BLAST) with high identity settings (Altschul et al., 1997) where the BLAST alignments that met all the criteria were considered products of candidate alternative splicing (AS) events (Liu et al., 2017): (1) the length of the two sequences is greater than 1,000 bp, and there are two High-scoring Segment Pairs in the alignment; (2) the AS Gap is larger than 100 bp and at least 100 bp away from the 3′/5′ end; and (3) allow a 5 bp “Overlap” size of all variable transcripts. Simple sequence repeats (SSR) of the transcriptome were identified using the MIcroSAtellite identification tool (MISA) (http://pgrc.ipk-gatersleben.de/misa/), while the TransDecoder (https://github.com/TransDecoder/TransDecoder/releases) identified the candidate coding regions within the transcript sequences.

### Gene functional annotation

Gene function was annotated based on the following databases: RefSeq non-redundant proteins (Nr) of The National Center for Biotechnology Information (NCBI), Protein family (Pfam), Gene Ontology (GO), Kyoto Encyclopedia of Genes and Genomes (KEGG), Swiss-Prot (a manually annotated and reviewed protein sequence database), and clusters of orthologous groups of proteins (KOG/COG/eggNOG) using E-value 10^−5^ as a cutoff.

### Illumina data processing and quantification of gene expression

Illumina data processing and quantification of gene expression was performed as previously described (Anders & Huber, 2010). Specifically, in-house Perl scripts were used to process the raw data. Clean reads were obtained by removing reads that contained adapters and poly-N and low-quality reads. For the quantification of gene expression level, the sum of the fragments mapped to each transcript was calculated. Then, the expression level was normalized. A differential expression analysis was performed using DESeq, where the screening criteria was a fold change ≥4 and a false discovery rate <0.05.

### Sequence analysis

We used the methods described in a previous report (Wolfgang et al., 2018) to obtain the homologous genes of *PqbZIP1* from *A. thaliana* as well as a BLAST search in the NCBI database. The sequence homology and conserved domains of these proteins were analyzed by the DNAMAN v6.0 (Lynnon Biosoft Corporation, San Ramon, CA, USA) and the NCBI Conserved Domain (CD)-Search (https://www.ncbi.nlm.nih.gov/Structure/cdd/wrpsb.cgi), while the Molecular Evolutionary Genetics Analysis (MEGA) v6.0 was used to build a phylogenetic tree using the Neighbor-Joining method. Finally, the Protein Subcellular Localization Prediction Tool (PSORT) (http://psort1.hgc.jp/form.html) was used to analyze the subcellular localization *in planta*.

### Expression profiles of *PqbZIP1* in different American ginseng tissues

The roots, stems, and leaves of a two-year-old healthy American ginseng plant were used for detecting the expression level of *PqbZIP1*, where each sample contained five replicates. The chitin and *F. solani*-treated roots were sampled at different time points after the treatment, with each time point including five individual plants. Total RNA of the different tissues and treated roots was extracted, after which the cDNA was synthesized. The expression of *PqbZIP1* was quantified using SYBY Premix Ex Taq II (Tli RnaseH Plus) (Takara, Japan), while the gene-specific primers (qPCR-*PqbZIP1*-F/qPCR-*PqbZIP1*-R) were designed using Primer v5.0, and *GAPDH* was used as a reference gene (qPCR-*GAPDH*-F/qPCR-*GAPDH*-R) (Table S12) (Chao et al., 2010). The qPCR was performed by the Bio-Rad CFX96 platform (Bio-Rad Laboratories, Inc., Hercules, CA, USA). Each sample was run in triplicates and repeated 3 times. Finally, the data was analyzed using the 2^−ΔΔCt^ method (Chen et al., 2015).

### Transactivation assays

The transactivation activity was analyzed as described previously (Gao et al., 2012): the *PqbZIP1* gene was amplified using Q-*PqbZIP1*-F /Q-*PqbZIP1*-R (Table S12) and then cloned into a pGBKT7 vector. The resulting pGBKT7-*PqbZIP1* fusion plasmids were then transformed into cells of the yeast-two-hybrid (Y2H) Gold strain (Clontech, Santa Clara, CA, USA). Strains introduced with the plasmids of pGBKT7-PCL-1 and the pGBKT7 empty vector served as positive and negative controls, respectively. The transformants were plated on YPDA, SD-Trp-His, and SD-Trp-His+X-a-gal media and then incubated at 30 °C for 2 days to determine the activation activity.

### Subcellular localization

The *PqbZIP1* gene was amplified using *PqbZIP1*-F /*PqbZIP1*-R (Table S12) and cloned into the pYBA1132 vector containing the green fluorescent protein (GFP). Then, the *PqbZIP1-GFP* fusion gene, or the vector control that only expressed GFP, were introduced into tobacco leaves through the agroinfiltration of GV3101. The infiltrated leaves were visualized at 48 h post inoculation (hpi) under a laser confocal fluorescence microscope (Zeiss LSM 880; Zeiss, Oberkochen, Germany) at an excitation wavelength of 488 nm (Chen et al., 2015).

### Growth ability of D36E strain

Either the *PqbZIP1-GFP* fusion gene or the vector control that only expressed GFP were introduced into tobacco leaves through the agroinfiltration of GV3101. After 48 hpi, *Pseudomonas syringae* pv. *tomato* D36E strain with a concentration of 1 × 10^8^ CFU/mL was infiltrated into the aforementioned leaves. *Pseudomonas syringae* pv. *tomato* D36E is a mutant strain of *P. syringae* pv. *tomato* DC3000 that knocks out 36 effectors which then stimulates the PAMP-triggered immunity response in plants (Wei, Zhang & Collmer, 2018). For each gene, five tobacco plants were infiltrated and three leaves of each tobacco plant were selected. After infiltrating for 4 days, we used a punch to sample a 0.9-cm diameter tobacco leaf, took a total of five points and combined them into one sample, added 500 μL of sterilized water, ground with a tissue grinder, and performed three treatments on each gene. Next, we took 100 μL of the above solution and diluted it by adding 900 μL of sterilized water. Then, we took 10 μL of the different concentration solutions of each treatment and added them dropwise to a 10-cm square petri dish. After the solution had dried, we covered the lid, repeated each gradient six times, and repeated each treatment three times, which were then cultured in a 28 °C incubator for 2 days, and then we counted the number of colonies.

### ROS

Luninol-HRP-based chemiluminescence assay was used to detect the ROS burst as previously described (Sang & Macho, 2017; Yang et al., 2019b). The *PqbZIP1-GFP* fusion gene, or the vector control that only expressed GFP, were introduced into tobacco leaves through the agroinfiltration of GV3101. At 36 hpi, leaf discs were collected and incubated in H_2_O overnight in a 96-hole whiteboard. Then, H_2_O was replaced by 100 μL elicitor master mix, and the plate was immediately put into the microplate reader for monitoring ROS production.

### Jasmonic acid, salicylic acid and abscisic acid content

The *PqbZIP1-GFP* fusion gene, or the vector control that only expressed GFP, were introduced into tobacco leaves through agroinfiltration of GV3101. After 48 hpi, the jasmonic acid, salicylic acid and abscisic acid content of the tobacco leaves was quantified by indirect enzyme-linked immunosorbent assay (ELISA) as previously described (Yang et al., 2001).

## Results

### Identification of strain 4171

Colony 4171 was rounded on the PDA and the hypha were white and villous (Fig. S1A), and it could cause severe root rot symptoms in American ginseng (Fig. S1B). Through the maximum likelihood tree of *Fusarium* spp. based on combined *ITS* and *tef-1α* genes, strain 4171 was determined to cluster with *Fusarium solani* (Fig. S1C). We named this strain 4171 “*Fusarium solani* 4171.”

### Combined sequencing of American ginseng transcripts

A dataset of 25.56 Gb clean reads was obtained, with a total of 360,700 circular consensus sequences (CCS) screened with full passes ≥3, and the sequence accuracy set at >0.9. The total number of CCS read bases was 619,830,996, whereas the mean read CCS length was 1,718 bp, and the mean number of passes was 47 (Table S1, Figs. S2A, S2B). The number of the full-length non-chimeric reads was 309,155 (85.71% of total CCS) (Table S2, Fig. S2C). In total, 145,855 consensus isoforms were obtained with an average read length of 1,719 bp, whereas there were 141,222 high-quality isoforms (96.82% of total consensus isoforms) (Table S3, Fig. S2D). As a result, we obtained 23,336,937; 21,901,311; 23,837,148; 24,351,979; 23,965,577; and 21,595,526 clean reads (Table S4).

### Analysis of AS, SSR, and CDS

The pre-mRNA, which is a precursor in eukaryotic gene transcription, has multiple splicing patterns; hence, when choosing different exons, different mature mRNAs are generated. Thus, the AS events were analyzed. In total, 3,132 AS events were found in American ginseng based on the PacBio SMRT reads (Table S5). We used MISA to analyze the transcripts, and seven types of SSRs were identified (Table S6). Based on the TransDecoder, 77,770 open reading frames (ORF) were obtained, where the number of complete ORF was 62,482. The predicted CDS can be seen in Table S7.

### Gene function annotation and categorization

**Figure 1:**
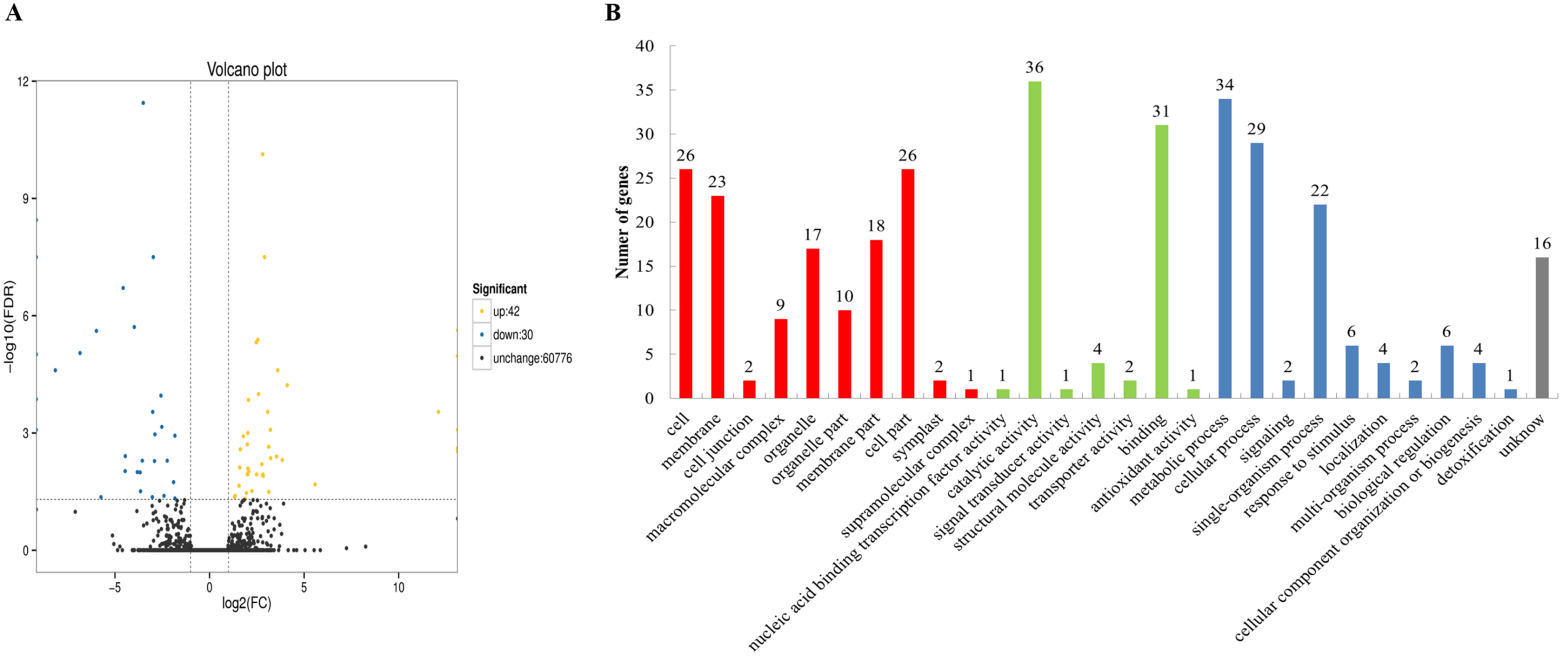
Volcano plot and Gene Ontology (GO) database of differential expressed genes (DEGs). (A) Volcano plot displaying differential expression between water- and chitin-treated American ginseng. The y-axis corresponds to the mean expression value of log (*P* value), and the x-axis displays the log2 fold-change value. The yellow dots represent transcripts whose expression was up-regulated, and the blue dots represent the transcripts whose expression was down-regulated. (B) GO annotation of the different expression transcripts of American ginseng affected by chitin.

To categorize the non-redundant transcripts, sequences were compared against Nr, Pfam, Swissprot, KEGG, GO, COG, KOG, and eggNOG. As a result, 77,901; 56,866; 58,168; 36,490; 62,023; 30,321; 52,732; and 76,376 transcripts were found to be annotated in the aforementioned databases, respectively (Table S8), with a total of 78,182 annotated transcripts. The databases Nr, Pfam, Swissprot, KEGG, GO, COG, KOG, and eggNOG distinguished the proteins by different domains. The GO terms described the functions of these cross-species homologous genes and their gene products, which were further divided into biological processes, cellular locations, and molecular functions. In this study, 72 differentially expressed genes were obtained between the chitin-treated and water-treated roots, among which 42 genes were upregulated, and 30 genes were downregulated (Fig. 1A). Among them 70 genes were annotated by COG, GO, KEGG, KOG, Pfam, Swiss-Prot, Swiss prot, eggNOG or nr database (Table S9). Some of the immune response related genes affected by chitin are listed in Table S10. The GO assignments of 51 subcategories within the three main categories are summarized in Fig. 1B, including the top three subcategories: catalytic activity, metabolic process, and binding.

### Analysis of transcription factors

**Figure 2:**
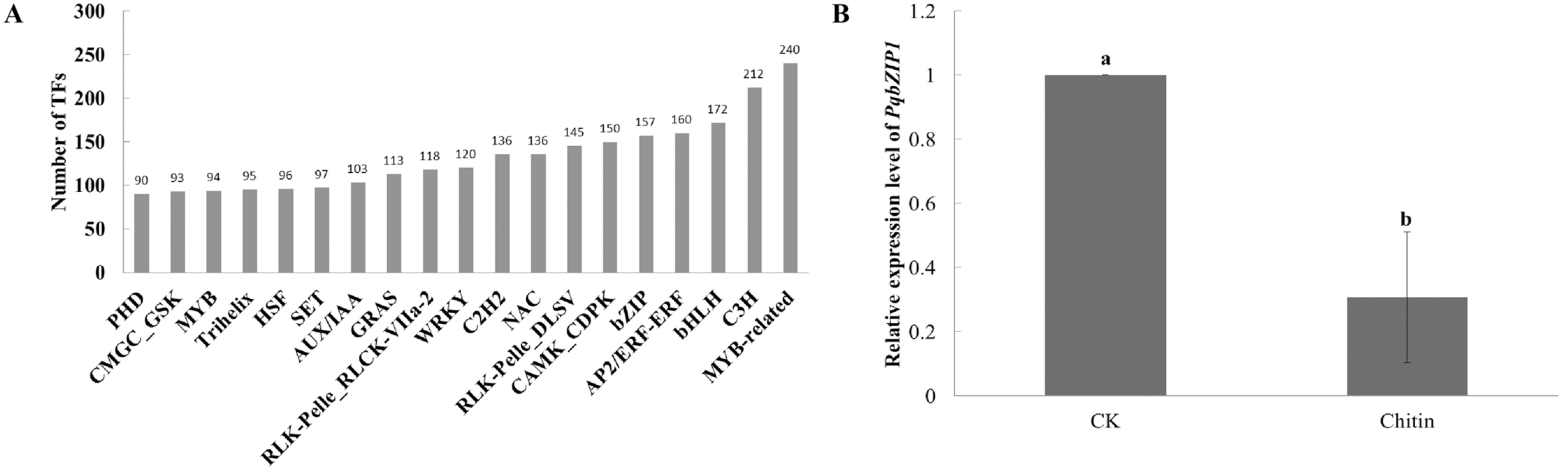
Transcription factors of American ginseng. (A) The transcription factors of American ginseng according to the full-length transcriptome and iTAK software. (B) The relative expression level of American ginseng *PqbZIP1* after being treated with chitin. Error bars represent standard error of the means. Bars with different letter represent results that are significantly different (*P* < 0.05, one-way ANOVA with Tukey’s multiple comparisons test). The experiment was repeated twice with similar results.

Transcription factors play important roles in plant growth and development as well as the resistance response (Chu et al., 2017). However, few transcription factors have been reported in American ginseng thus far. To analyze the transcription factors in American ginseng, the iTAK software (Zheng et al., 2016) was used. As a result, 6,813 transcription factors were identified, which were classified into 209 families (Table S11); among them, the MYB-related, C3H, bHLH, AP2/ERF-ERF, and bZIP were the top five families classified followed by CAMK_CDPK, RLK-Pelle DLSV, NAC, and C2H2 (Fig. 2A). Through analyzing the differentially expressed genes between the water and chitin-treated samples of American ginseng, we found a transcription factor (named PqbZIP1) that was significantly induced by chitin treatment. The expression level of *PqbZIP1* was validated by qPCR (Fig. 2B).

### Sequence analysis of *PqbZIP1* from American ginseng

**Figure 3:**
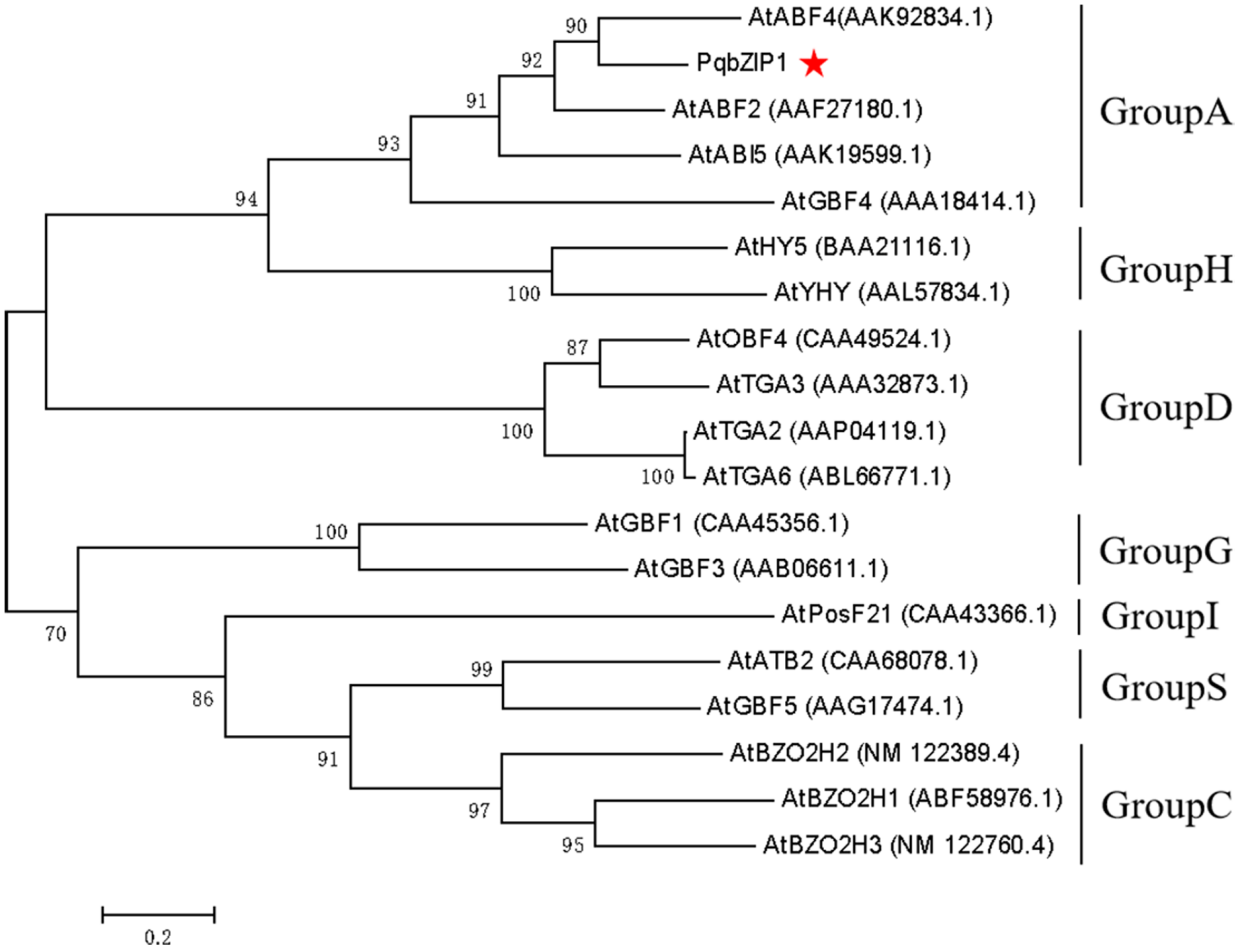
Phylogenetic tree for PqbZIP1 and its homologs from *A. thaliana*.

The *PqbZIP1* cDNA was found to encode a 456-amino acid protein with a predicted molecular size of 50.52 kDa including a bZIP conserved domain (Fig. S3A). PSORT analysis showed that *PqbZIP1* had a nuclear localization (Fig. S3B). In terms of the alignment analysis, the homologs with the highest similarity to Ntphi-2 from *N. benthamiana*, which responded to abscisic acid treatment (Sano & Nagata, 2002), showed 48.35% similarity. According to the phylogenetic tree, PqbZIP1 and three proteins (AtABF2, AtABI5, AtGBF4 and AtGBF4) of *A. thaliana* were clustered together (Fig. 3), which was a part of group A of the bZIP family in *A. thaliana* (Wolfgang et al., 2018).

### Expression profiles of *PqbZIP1*

**Figure 4:**
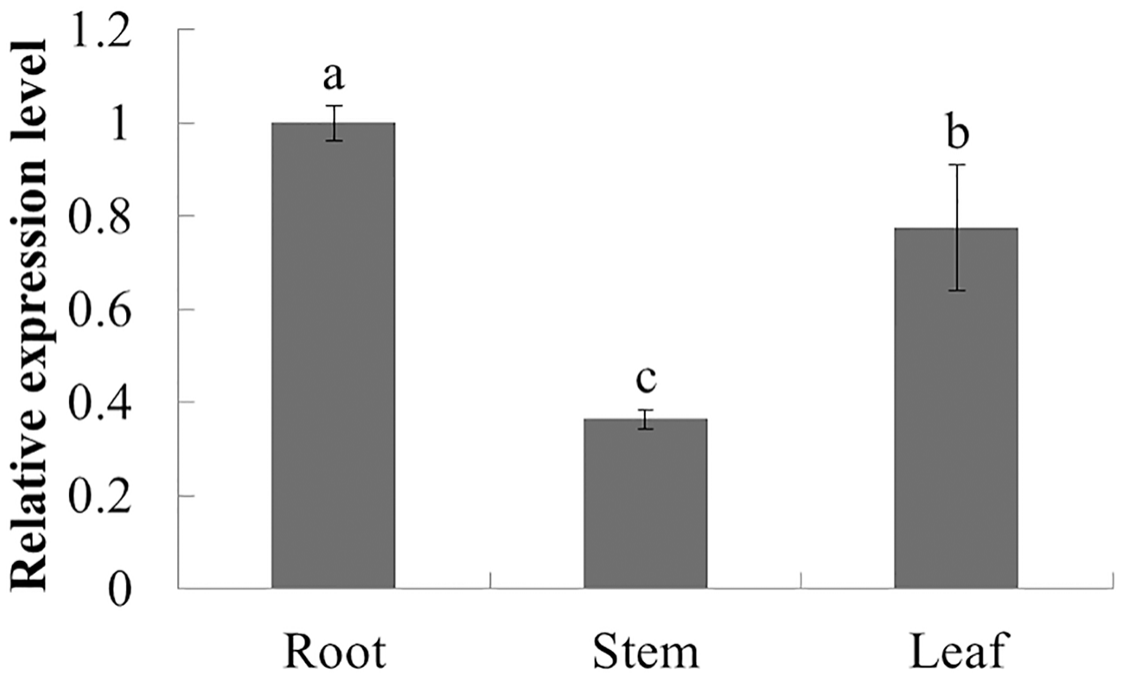
Relative expression level of *PqbZIP1* in different tissues of American ginseng. Different letters indicate statistically significant differences (*P* < 0.05, one-way ANOVA with Tukey’s multiple comparisons test). The error bars represent standard error of the means. The experiment was repeated three times with similar results.

The expression characteristics of the target gene in different tissues of American ginseng were analyzed by qPCR. The results showed that *PqbZIP1* could be expressed in different tissues and vary significantly. *PqbZIP1’s* expression level was the highest in the roots, followed by the leaves, whereas the stems had the lowest level of expression; the expression level of *PqbZIP1* in the roots was 2.8 times higher than that in the stems (Fig. 4). It is hypothesized that *PqbZIP1* might play an important role in the growth and development of roots and leaves.

**Figure 5:**
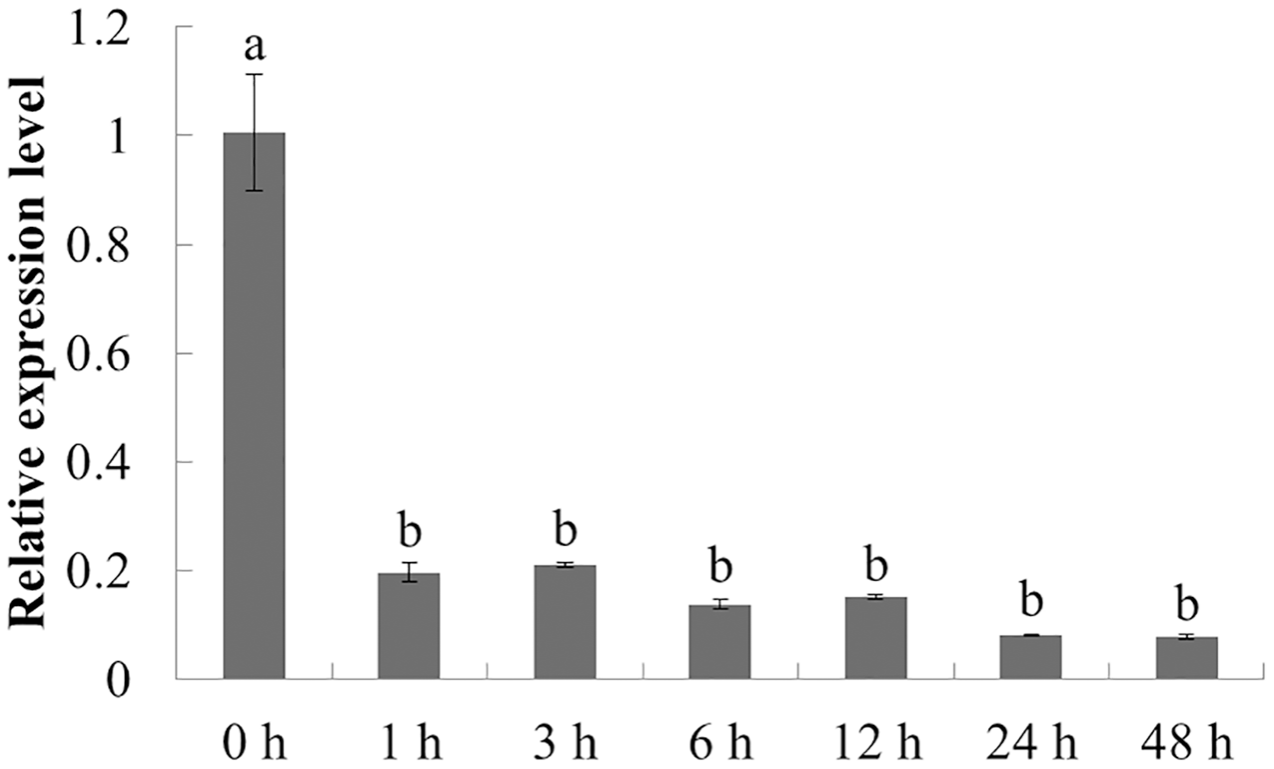
Transcriptional profiling of *PqbZIP1* in response to chitin treatment. Different letters indicate statistically significant differences (*P* < 0.05, one-way ANOVA with Tukey’s multiple comparisons test). The error bars represent standard error of the means. The experiment was repeated three times with similar results.

The expression characteristics of the target gene in American ginseng at different periods after chitin treatment were analyzed by qPCR. As shown in Fig. 5, the expression of *PqbZIP1* was significantly inhibited at 1 h post treatment (hpt), showing an 80.5% decline, and reached its lowest level at 24 hpt. This showed that chitin could significantly inhibit the expression of *PqbZIP1*.

**Figure 6:**
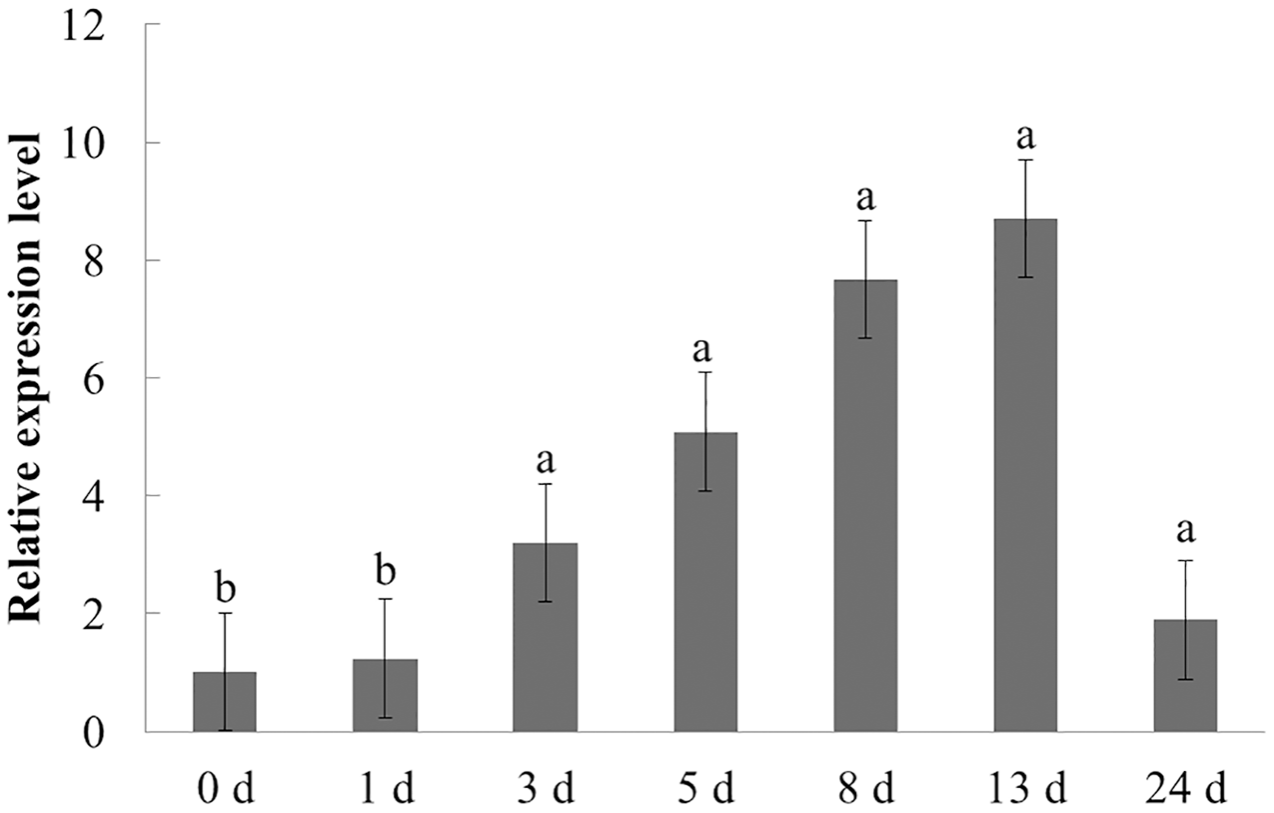
Transcriptional profiling of *PqbZIP1* in response to *F. solani* infection. Different letters indicate statistically significant differences (*P* < 0.05, one-way ANOVA with Tukey’s multiple comparisons test). The error bars represent standard error of the means. The experiment was repeated three times with similar results.

The expression characteristics of the target gene in American ginseng at different periods after *F. solani* inoculation were then analyzed by qPCR. As shown in Fig. 6, *F. solani* and chitin had opposing effects on the expression level of *PqbZIP1* in American ginseng. *F. solani* could significantly stimulate the expression of *PqbZIP1* with extension of the inoculation time, showing an increase of 7.7- fold at 13 hpi. This showed that *F. solani* could significantly increase the expression of the *PqbZIP1* transcription factor, which played an important role in the disease resistance of American ginseng.

### Transcriptional activity of PqbZIP1

**Figure 7:**
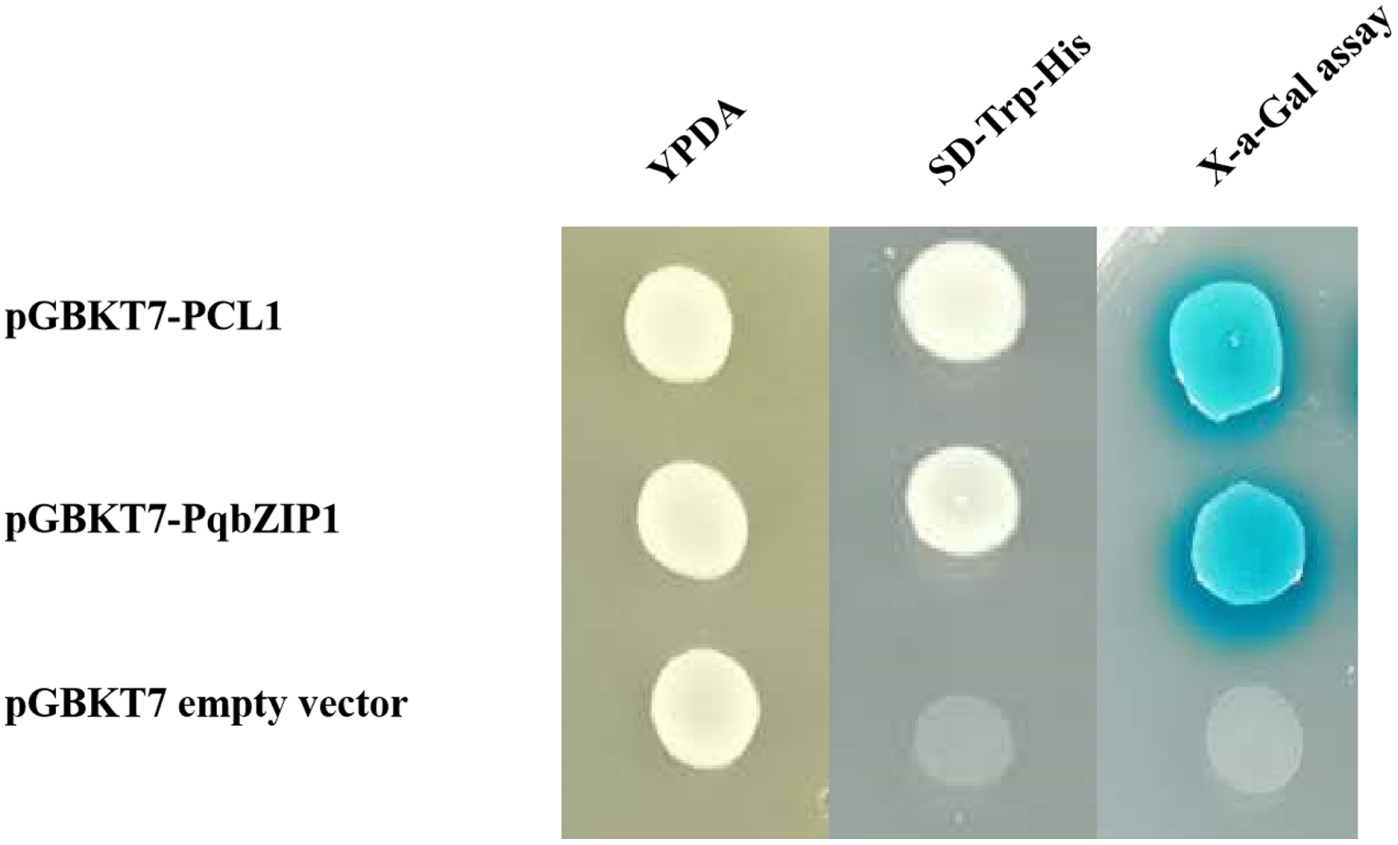
Transactivation assay of PqbZIP1. The yeast-two-hybrid (Y2H) Gold strain expressing pCL1, BD, and PqbZIP1-BD grew on YPDA or the selective medium SD-Trp-His. The pCL1 encoding the full-length GAL4 and the empty vector pGBKT7 (BD) were used as positive and negative controls, respectively.

Transcriptional activity is an important index to evaluate transcription factors. The pGBKT7-*PqbZIP1* plasmid was transformed into the yeast strain Y2H to determine the transcriptional activity of *PqbZIP1*. The Y2H Gold yeast was transformed with pGBKT7-*PqbZIP1*, pGBKT7-PCL1, or the pGBKT7 empty vector, which resulted in growth on yeast extract peptone dextrose media, indicating that PqbZIP1 had no toxicity to the Y2H Gold yeast. The Y2H Gold yeast transformed with pGBKT7-*PqbZIP1* or pGBKT7-PCL1 grew normally in a double-deficient media, whereas the negative control yeast that was transformed with the pGBKT7 empty vector did not grow (Fig. 7), which indicated that the PqbZIP1 protein was transcriptionally active.

### Subcellular localization of PqbZIP1

**Figure 8:**
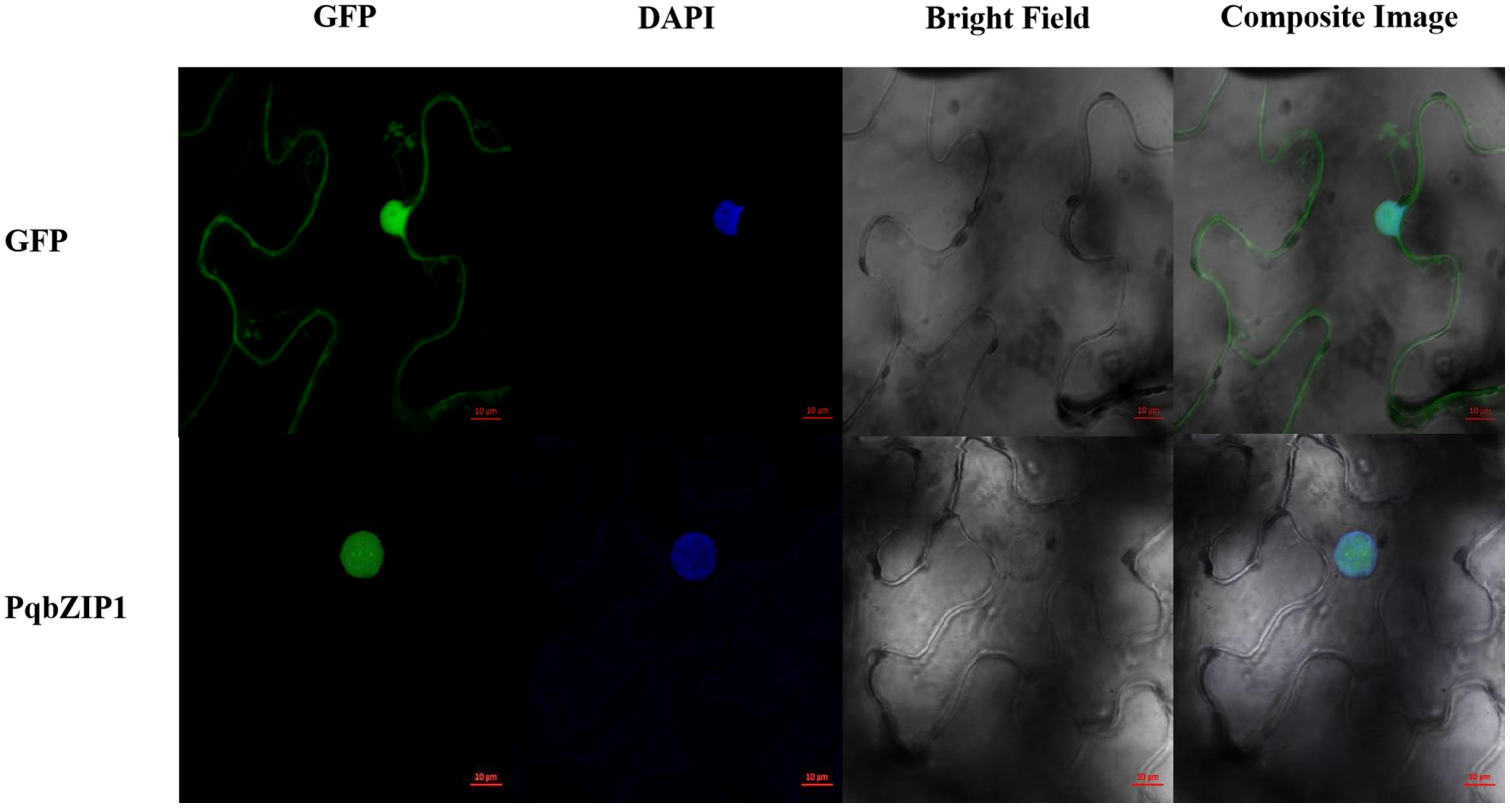
Subcellular location of PqbZIP1 of American ginseng. The subcellular location experiment of PqbZIP1 revealed that PqbZIP1-GFP was located in the nucleus of *N. benthamiana* leaves, while GFP was detected throughout the whole cell. Scale bar = 20 mm.

A typical transcription factor is often located in the nucleus of plant cells (Schwechheimer, Zourelidou & Bevan, 1998). The results showed that GFP fluorescence was only detected in the nucleus of PqbZIP1-GFP fusion protein-infiltrated tobacco cells, whereas the GFP fluorescence was evenly distributed throughout the tobacco cells expressing GFP (Fig. 8). Taken together, these results indicated that PqbZIP1 was localized in the plant nucleus and was a typical transcription factor.

### PqbZIP1 could inhibit the growth ability of D36E in *N. benthamiana*

**Figure 9:**
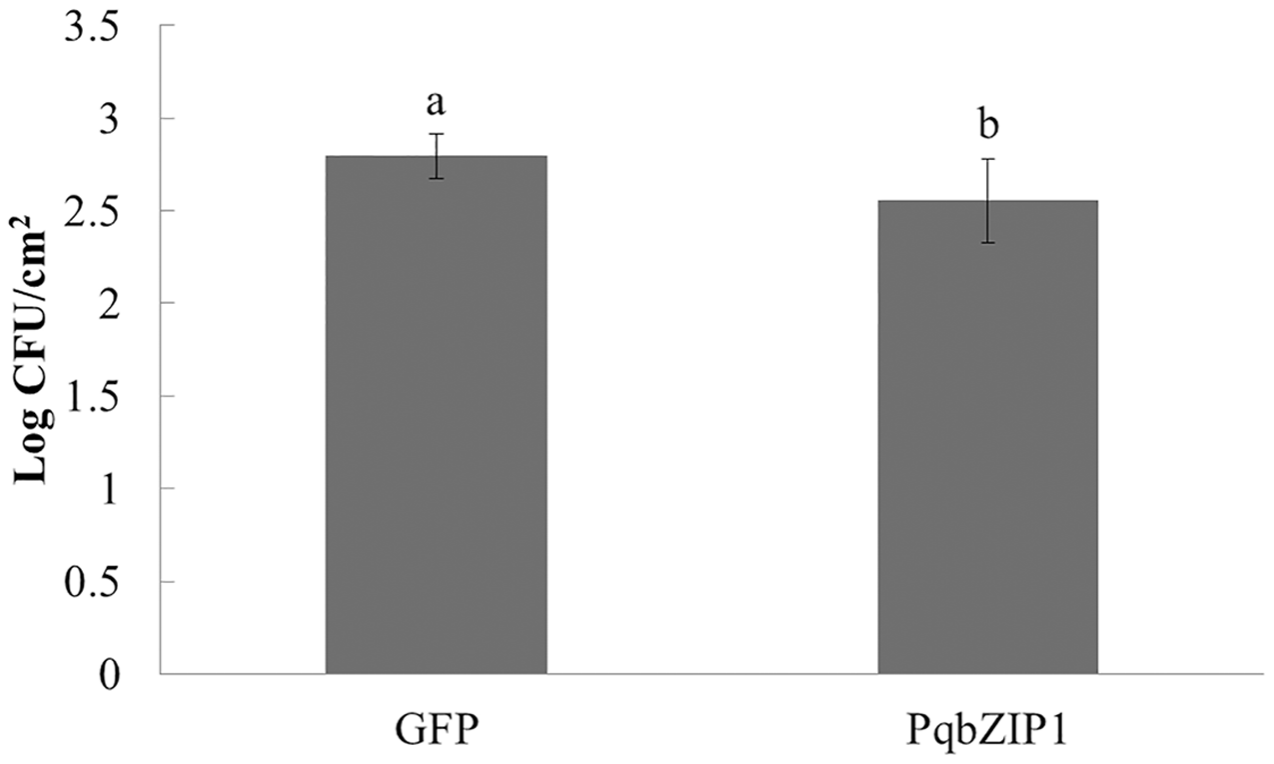
PqbZIP1 increased the growth ability of D36E in *N. benthamiana*. Different letters indicate statistically significant differences (*P* < 0.05, one-way ANOVA with Tukey’s multiple comparisons test). The error bars represent standard error of the means. The experiment was repeated twice with similar results.

The aforementioned experiments showed that PqbZIP1 was a transcription factor and its expression was affected by chitin and *F. solani*. We then detected its effect on pathogen invasion through heterologous expression in plants. Before infiltrating the D36E strain, PqbZIP1-GFP or GFP alone were expressed in the leaves of tobacco. By quantifying D36E colonies, the function of *PqbZIP1* could be identified, where the number of D36E colonies prior to being injected with GV3101 containing PqbZIP1-GFP was significantly fewer than that prior to injection with GV3101 containing GFP, showing a 36.8% decline (Fig. 9). These results showed that PqbZIP1 significantly inhibited the growth ability of D36E in *N. benthamiana* and could participate in the plant’s disease resistance response. As the expression of *PqbZIP1* could be stimulated by *F. solani*, we hypothesized that it could also play a role in the resistance response of American ginseng and inhibit the infection of *F. solani*.

### PqbZIP1 could advance the burst time of active oxygen

**Figure 10:**
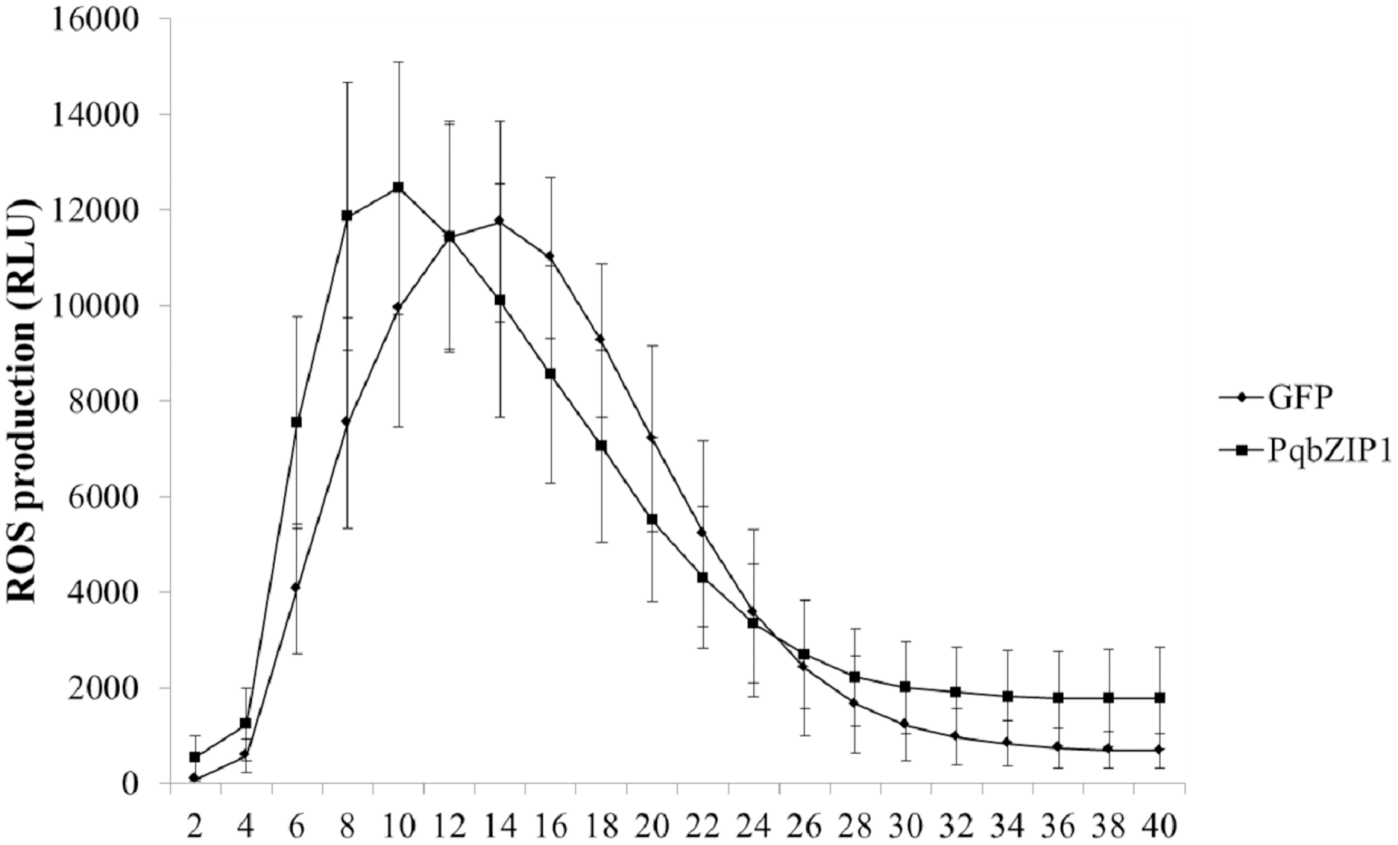
*Agrobacterium tumefaciens* strain GV3101 carrying PqbZIP1-GFP advanced the burst time of active oxygen induced by flg22 in *Nicotiana benthamiana* compared with that carrying GFP. Different letters indicate statistically significant differences (*P* < 0.05, one-way ANOVA with Tukey’s multiple comparisons test). The error bars represent standard error of the means. The experiment was repeated twice with similar results.

The ROS burst was a hallmark event of PTI response; therefore, we investigated whether PqbZIP1 could influence the ROS production induced by flg22. The PqbZIP1-GFP fusion gene, together with the vector control expressing GFP only, was introduced into tobacco leaves through agroinfiltration. At 36 hpi, leaf discs were collected and treated with flg22 using the previously described luminol-based method (Sang & Macho, 2017). The results showed that PqbZIP1 advanced the burst time of active oxygen by 4 min (Fig. 10).

### PqbZIP1 could increase jasmonic acid, salicylic acid and abscisic acid content

**Figure 11:**
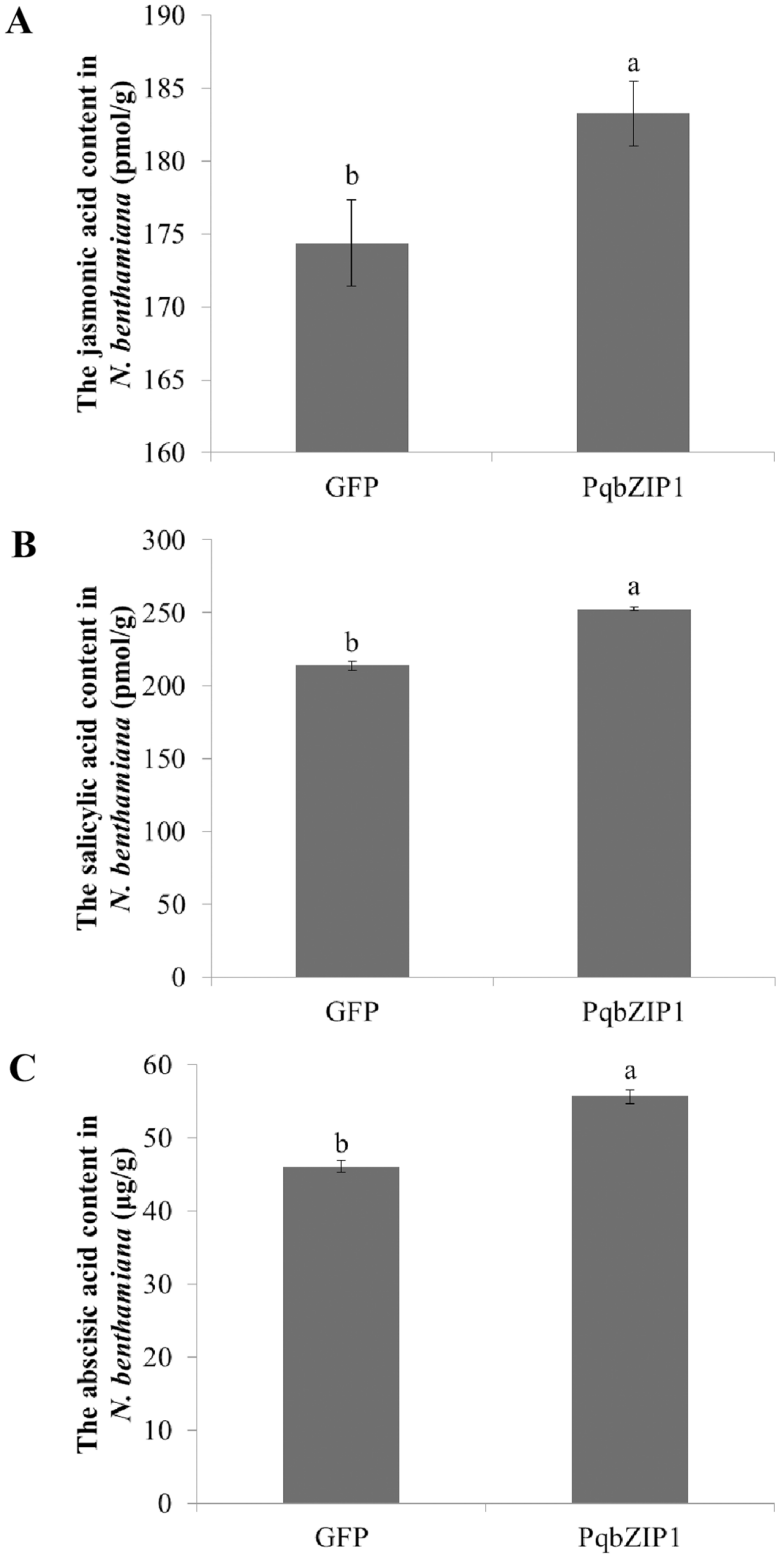
PqbZIP1 increased plant jasmonic acid (A), salicylic acid (B) and abscisic acid (C) content. Different letters indicate statistically significant differences (*P* < 0.05, one-way ANOVA with Tukey’s multiple comparisons test). The error bars represent standard error of the means. The experiment was repeated three times with similar results.

To further explore the cause of *PqbZIP1* expression induction by *F. solani*, and the reason *PqbZIP1* could inhibit the growth of D36E in *N. benthamiana*, we analyzed the hormonal content of *N. benthamiana* expressing *PqbZIP1*. As shown in Fig. 11A, the jasmonic acid content in the *N. benthamiana* expressing *PqbZIP1* for 48 h was significantly higher than the jasmonic acid content when expressing GFP, showing an increase of 5.1%. The salicylic acid content in the *N. benthamiana* expressing *PqbZIP1* for 48 hpi was significantly higher than the salicylic acid content when expressing GFP, showing an increase of 18.2% (Fig. 11B). Further, the *N. benthamiana* expressing *PqbZIP1* for 48 hpi was significantly higher than that when expressing GFP, showing an increase of 20.8% (Fig. 11C). All these results showed that PqbZIP1 could significantly increase jasmonic acid, salicylic acid and abscisic acid content in *N. benthamiana*.

## Discussion

As genomic information regarding American ginseng was not yet available, we sequenced the full-length transcriptome and analyzed the gene expression profile of American ginseng in this study. In addition, the differentially expressed transcriptome was used to explore the genes associated with plant immune response. Thus, American ginseng was treated with chitin to explore the differentially expressed genes. As a component of fungal cell walls, chitin can stimulate the plant immune response. Compared with the complexity of *F. solani*, treatment with chitin was an effective method for mining the resistance genes of American ginseng. To the best of our knowledge, this was the first instance of using chitin treatment to identify the immune-related genes of American ginseng. Studying the functions of these genes was important in guiding the prevention and control of fungal diseases in American ginseng. As expected, many American ginseng genes were induced by chitin (Fig. 1), and some of these were immune response related genes (Table S10).

In plants, bZIP transcription factors regulate many processes, including pathogen defense, light and stress signaling, seed maturation, and flower development. According to the domains shared by the bZIP proteins, 13 groups of bZIPs (designated A-M) were obtained (Wolfgang et al., 2018; Jakoby et al., 2002; Singh, Foley & Oñate-Sánchez, 2002; Alves et al., 2013; Liu et al., 2014; Lim et al., 2015; Li et al., 2017; Liu et al., 2018; Wang et al., 2019; Yang et al., 2019a; Yu et al., 2019). In this assay, in view of the important role of transcription factors in the immune response of plants, the transcription factor PqbZIP1 was selected for further research to explore its relationship with plant immunity. The phylogenetic tree showed that *PqbZIP1* belonged to group A of bZIPs (Fig. 3). According to the existing reports, the group A members of bZIP transcription factors participated in ABA signaling. For example, group A members played roles in abscisic acid or stress signaling in *A. thaliana* (Uno et al., 2000; Finkelstein & Lynch, 2000; Choi et al., 2000). The group A members performed important functions in ABA signaling, and they were associated with abiotic stresses in carrots (Que et al., 2015). However, in our study, we found that expressing *PqbZIP1* in *N. benthamiana* could not only increase the abscisic acid content, but also the jasmonic acid and salicylic acid content (Fig. 11). These results confirmed that the content of abscisic acid, jasmonic acid, and salicylic acid were regulated by *PqbZIP1* transcription factors. From the qPCR experiment, we knew that *F. solani* could activate the expression of *PqbZIP1* in American ginseng. It was well known that hormone signaling pathways played a very important role in the process of plant disease resistance (Pieterse et al., 2012; Dong, 1998). Therefore, we speculated that the PqbZIP1 transcription factor could mediate the jasmonic acid, salicylic acid, and abscisic acid signaling pathways to modulate root rot disease resistance in American ginseng, which was a new discovery. This is the first report showing the involvement of the PqbZIP1 transcription factor in the jasmonic acid and salicylic acid signaling pathways, but further study is needed to identify the associated mechanisms.

The PqbZIP1 transcription factor was screened with chitin treatment, which only indicated that PqbZIP1 was induced by fungi. However, when faced with *F. solani*, its real expression pattern required experimental validation. From the qPCR experiment, we found that chitin could inhibit the expression of *PqbZIP1* (Fig. 5), whereas *F. solani* activated the expression of *PqbZIP1* in American ginseng (Fig. 6). At first glance, this result seemed to be contradictory, but when compared with chitin, which was the conserved component of fungal cell walls, the fungi were much more complex. Fungi had evolved various strategies to dampen the chitin-triggered immunity in plants by blocking either the chitin perception or its signal transduction (Gong, Wang & Li, 2020). First, many fungal pathogens encoded apoplastic effectors that contain the LysM domain to outcompete the plant chitin receptors that harbor the same domain (Yoshida et al., 2010). Second, fungal pathogens also employed non-LysM effectors to protect their cell walls or to interfere with plant chitin perception (Stergiopoulos et al., 2010). Third, many fungi encoded chitin or polysaccharide deacetylases to convert chitin to chitosan (Xu et al., 2020). Chitin also inhibited the expression of *PqbZIP1*, which could only be explained by the PqbZIP1 transcription factor being related to the immune response of American ginseng, but the relationship requires further experimental analysis. Hence, in this study, we sequenced the transcriptome of American ginseng and identified the transcription factor PqbZIP1, which participated in multiple hormone signaling pathways to modulate resistance against root rot disease in American ginseng. However, further research is required to understand its target and how it regulates multiple hormone signaling pathways.

## Conclusions

This was the first instance of using chitin treatment to identify the immune-related genes of American ginseng. The differentially expressed transcriptome was used to explore the genes associated with the plant’s immune response. Our study made a significant contribution to the literature because genomic information regarding American ginseng was not yet available; hence, we sequenced the full-length transcriptome and analyzed the gene expression profile of American ginseng in this study. Finally, we screened immune-related transcription factor PqbZIP1, and found it might mediate multiple hormone signaling pathways to modulate root rot disease resistance in American ginseng. This study provides important information in guiding the prevention and control of fungal diseases in American ginseng.

## Supplemental Information

10.7717/peerj.12939/supp-1 Supplemental Information 1 Identification of *F. solani*. (A) The morphology of *F. solani* on PDA plate. (B) Symptoms of American ginseng root after inoculation 10 days. (C) Maximum likelihood tree of *Fusarium* spp. based on combined of *ITS* and *tef-1α* genes.

10.7717/peerj.12939/supp-2 Supplemental Information 2 Library construction of PacBio sequencing. (A) Circular consensus sequencing (CCS) read length distribution of each size bins. (B) Distribution of full passes generating CCS sequence. (C) Full-length reads non-chimeric distribution of each size bin. (D) Consensus isoforms read length distribution.

10.7717/peerj.12939/supp-3 Supplemental Information 3 The conserved domain and subcellular location analysis of PqbZIP1. (A) The conserved domain of PqbZIP1. (B) Subcellular location prediction of PqbZIP1.

10.7717/peerj.12939/supp-4 Supplemental Information 4 Sequence analysis of bZIP transcription factor genes from American ginseng. The nucleic acid sequences of F01_transcript_129280 and F01_transcript_3374 have 99% similarity

10.7717/peerj.12939/supp-5 Supplemental Information 5 Statistics of sequencing data for the single molecule, real-time (SMRT) cell by PacBio sequel II sequencing.

10.7717/peerj.12939/supp-6 Supplemental Information 6 Full-length sequence data statistics.

10.7717/peerj.12939/supp-7 Supplemental Information 7 Clustering result statistics.

10.7717/peerj.12939/supp-8 Supplemental Information 8 Sample sequencing data evaluation statistics.

10.7717/peerj.12939/supp-9 Supplemental Information 9 Alternative splicing analysis of transcript sequences.

10.7717/peerj.12939/supp-10 Supplemental Information 10 Simple sequence repeat (SSR) analysis of transcript sequences.

10.7717/peerj.12939/supp-11 Supplemental Information 11 Coding sequences (CDS) of transcript sequences.

10.7717/peerj.12939/supp-12 Supplemental Information 12 Transcript function notes.

10.7717/peerj.12939/supp-13 Supplemental Information 13 The differentially expressed genes affected by chitin.

10.7717/peerj.12939/supp-14 Supplemental Information 14 Some of the immune response related genes induced by chiti.

10.7717/peerj.12939/supp-15 Supplemental Information 15 Transcription factors in American ginseng.

10.7717/peerj.12939/supp-16 Supplemental Information 16 Primers used in this study.

10.7717/peerj.12939/supp-17 Supplemental Information 17 Raw Data.

## Additional Information and Declarations

### Data Availability

The following information was supplied regarding data availability: The transcriptome datas of American ginseng treated by water were SRR13447963 (T01), SRR13447962 (T02) and SRR13447961 (T03); the transcriptome datas of American ginseng treated by chitin were SRR13447967 (T07), SRR13447966 (T08), SRR13447965 (T09); and the transcriptome data of the mixture of water treatment and chitin treatment American ginseng roots was SRR13447964 (F01).
