# Supplementary figures and images for: A bZIP transcription factor, PqbZIP1, is involved in the plant defense response of American ginseng
Source: PeerJ. 2022 Mar 8;10:e12939. doi: 10.7717/peerj.12939 (PMC8916028; doi:10.7717/peerj.12939)

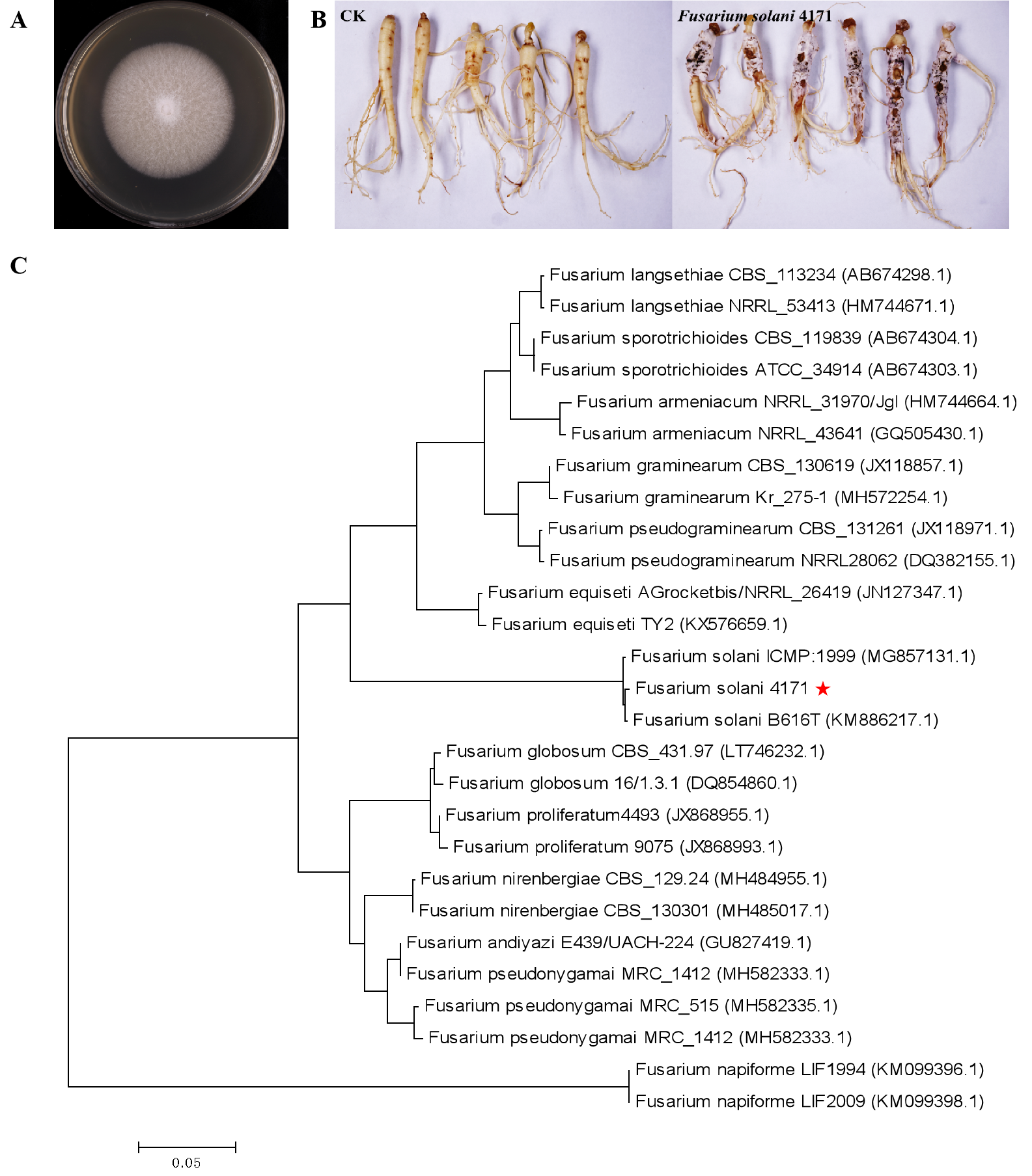

Supplement: Supplemental Information 1 — (A) The morphology of F. solani on PDA plate. (B) Symptoms of American ginseng root after inoculation 10 days. (C) Maximum likelihood tree of Fusarium spp. based on combined of ITS and tef-1α genes. [file peerj-10-12939-s001.png]

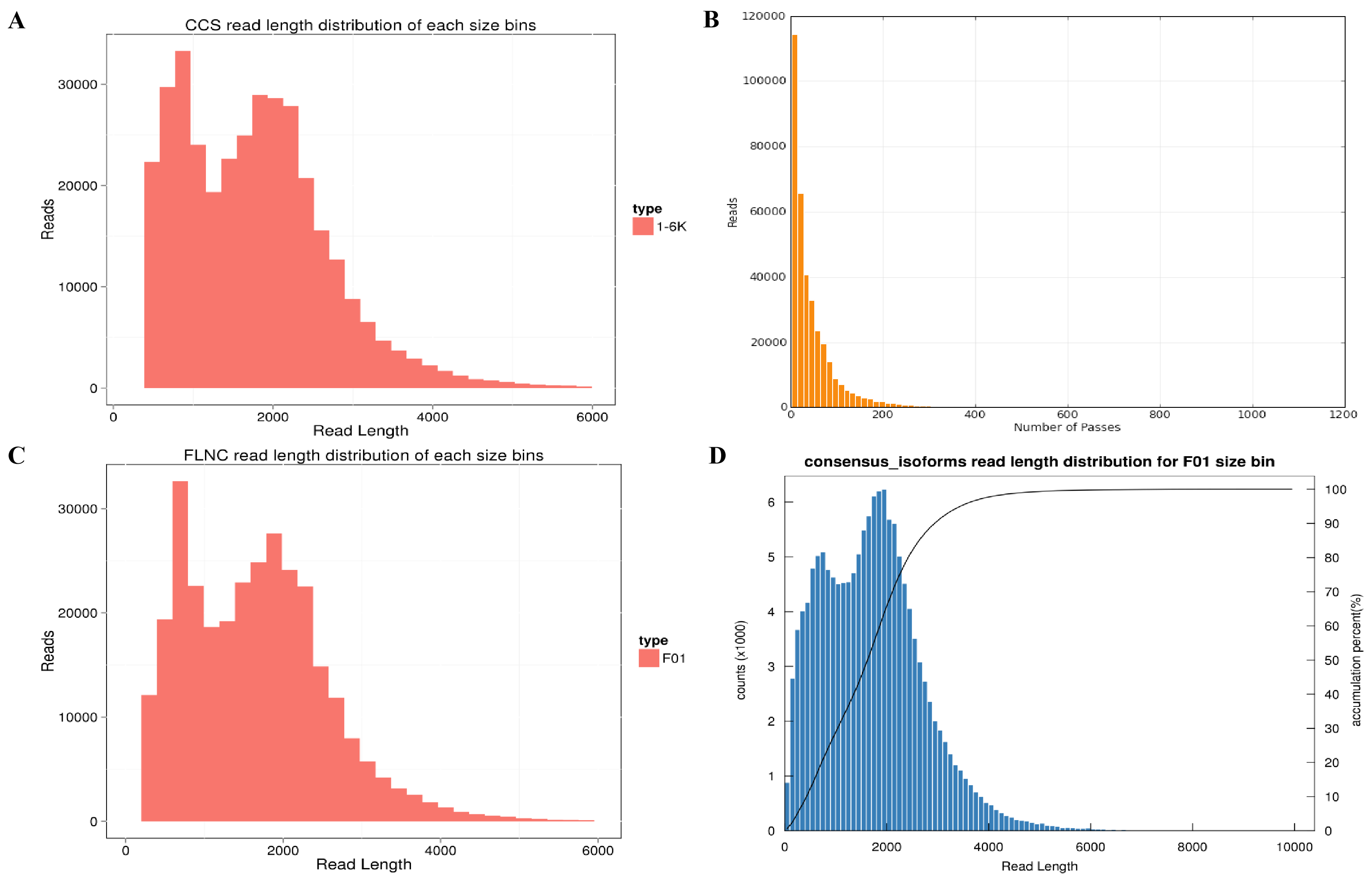

Supplement: Supplemental Information 2 — (A) Circular consensus sequencing (CCS) read length distribution of each size bins. (B) Distribution of full passes generating CCS sequence. (C) Full-length reads non-chimeric distribution of each size bin. (D) Consensus isoforms read length distribution. [file peerj-10-12939-s002.png]

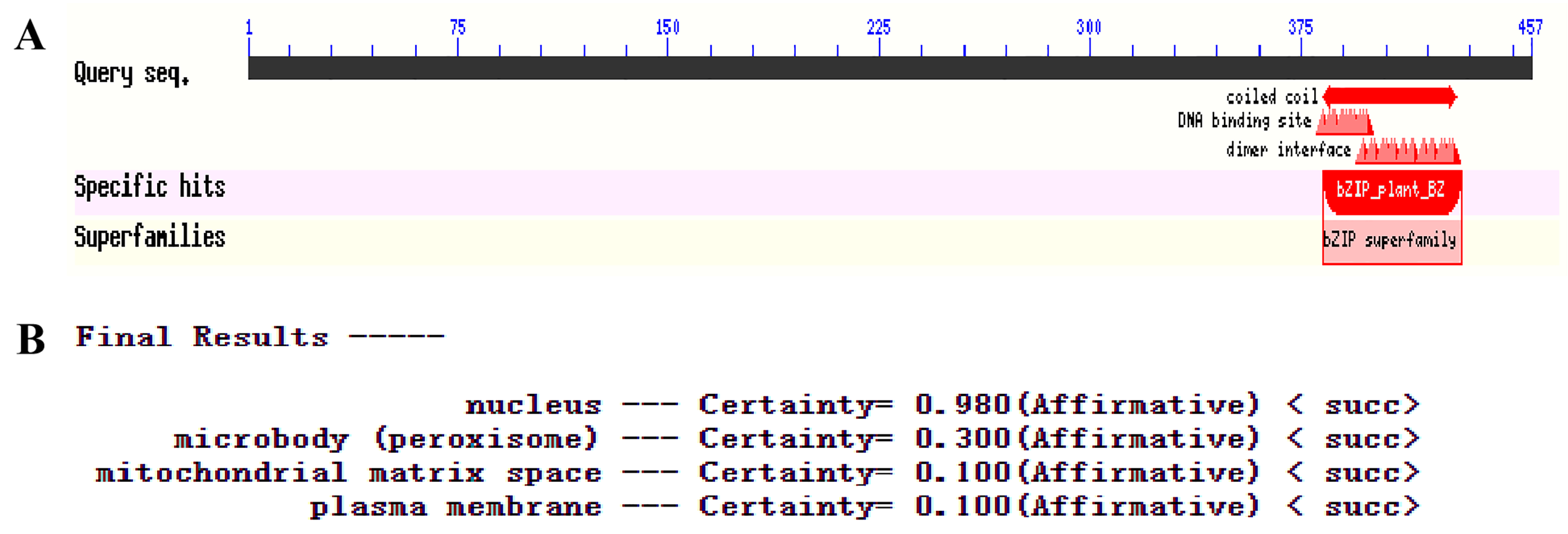

Supplement: Supplemental Information 3 — (A) The conserved domain of PqbZIP1. (B) Subcellular location prediction of PqbZIP1. [file peerj-10-12939-s003.png]

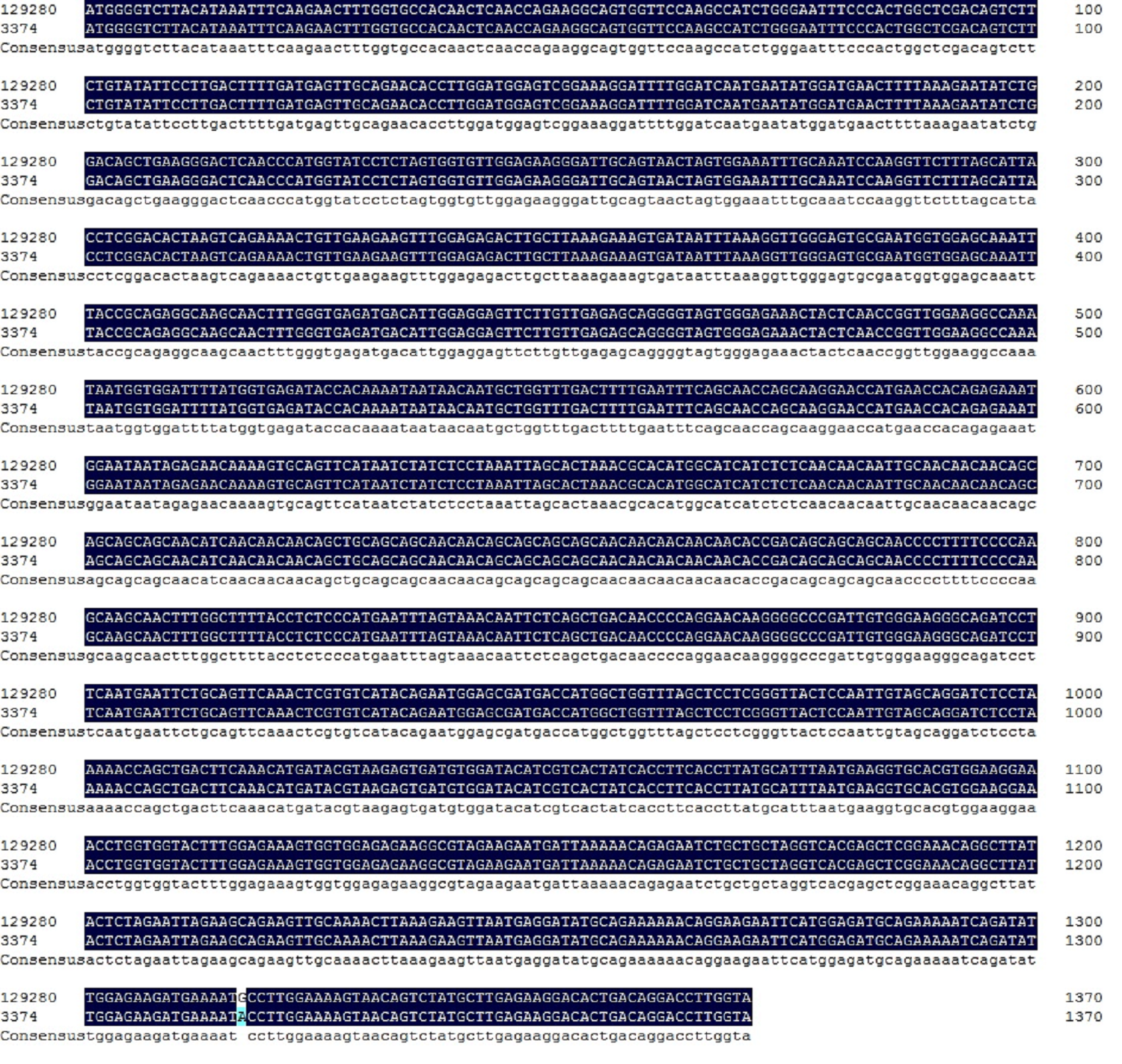

Supplement: Supplemental Information 4 — The nucleic acid sequences of F01_transcript_129280 and F01_transcript_3374 have 99% similarity [file peerj-10-12939-s004.png]
